# Beginner’s Intelligibility Test (BIT): translation, cultural adaptation to Brazilian Portuguese and validation^☆^

**DOI:** 10.1016/j.bjorl.2023.101311

**Published:** 2023-08-23

**Authors:** Carla Dias da Silva, Nelma Ellen Zamberlan-Amorim, Myriam de Lima Isaac, Ana Cláudia Mirândola Barbosa Reis

**Affiliations:** a Sao Paulo of University, Ribeirão Preto Medical School, Graduate Program in Rehabilitation and Functional Performance, Ribeirão Preto, SP, Brazil; b University of São Paulo at Ribeirão Preto, College of Nursing, Program Public Health Nursing, Ribeirão Preto, SP, Brazil; c University of Sao Paulo, Graduate Program Ophthalmology, Otorhinolaryngology, Head and Neck Surgery, Ribeirão Preto, SP, Brazil; d University of São Paulo, Ribeirão Preto Medical School, Sciences Health Department, Ribeirão Preto, SP, Brazil

**Keywords:** Tests, Speech intelligibility, Child, Hearing impairment

## Abstract

**Objective:**

Translate and validate the Beginner's Intelligibility Test (BIT) speech intelligibility assessment instrument into Brazilian Portuguese.

**Method:**

Study developed in two stages: 1st translation and cross-cultural adaptation of the Beginner’s Intelligibility Test instrument into Brazilian Portuguese; 2nd application of the instrument. The second stage of the study involved 20 children using cochlear implants, aged between 4 and 11 years old, enrolled in the Cochlear Implant Program at Hospital das Clínicas de Ribeirão Preto, Faculty of Medicine of Ribeirão Preto, University of São Paulo. All data collection procedures were video, and audio recorded for analysis by judges. After orthographic transcription of each sentence repeated by the child, the judge was asked to score the participant’s speech intelligibility and classify it according to criteria established in the literature.

**Results:**

The translation stage took place for the four lists and 40 sentences of the Beginner’s Intelligibility Test (BIT) instrument into Brazilian Portuguese, the semantic, idiomatic, experimental and conceptual equivalences were considered, and it took place without any difficulties identified by the translators. The terms used in the Portuguese language were similar and those that presented differences among the translators did not bring significant divergences to its understanding. In the inter-evaluator analysis, there was reliability between the classification and the score obtained. Different judges evaluated the same children and a concordance was observed in classification and scoring.

**Conclusions:**

Face validity of the BIT was confirmed through the understanding of each sentence of the four lists by the majority of children using cochlear implants participating in the pre-test phase. The content validity among experts was unanimous for the four lists of sentences. The Brazilian Portuguese adapted version maintained the semantic, idiomatic, conceptual, and cultural equivalence, according to the evaluation of the expert committee.

**Evidence level:**

02.

## Highlights

• BIT can be useful for assessing speech intelligibility of children.
• BIT can be applied in the evaluation, follow-up and intervention.
• The translated and adapted version of the BIT showed good reliability parameters.

## Introduction

Speech intelligibility can be defined as the accuracy with which a speaker can produce speech that is understandable by other listeners. This is an important and difficult skill to be developed in children with profound Hearing Deficiency (HD).^1^, ^2^ In children with HD it has been studied over the years, with special interest from parents and professionals, since the results regarding intelligibility provide guidance on the feasibility of oral communication in these children.^3^

Despite the literature highlighting that, as the time of use of the electronic device increases, the results are better,^4^ it is important to emphasize that there are variables that can potentially contribute to the variability of children’s performance using CI, such as: the child’s age at the time of surgery, time of sensory deprivation, time of CI usage, degree of family participation in the therapeutic process, type of CI and speech coding strategy.^5^, ^6^, ^7^

According to Chin et al.,^8^ among the main speech materials developed to assess speech intelligibility, the Beginner’s Intelligibility Test (BIT) stands out, proposed by Osberger et al. which is a research instrument developed to assess the speech intelligibility of children with cochlear implants, using the transcription procedure. The BIT test consists of four lists of sentences with a simple grammatical structure and with words familiar to the child. All words are made up of monosyllable to polysyllable words. Each sentence contains between 2 and 6 words (average = 3.8 words) and between 4 and 10 syllables (average = 6.5 syllables). All lists contain 40 words, with words been scored equally.

Studies on the speech intelligibility of children who use electronic hearing aid devices, as well as the need for instruments adapted and validated for the local culture, provide crucial information about the child's performance at the time of evaluation, follow-up and therapeutic planning and intervention.

Thus, the present work aimed at the translation and cross-cultural adaptation of the Beginner’s Intelligibility Test (BIT)^9^ instrument.

## Material and methods

Observational, prospective, cross-sectional study. Approved by the Ethics Committee of HCFMRP-USP under opinion number 1.909. This research followed the translation and cross-cultural adaptation recommendations according to the main research in the area.^10^, ^11^, ^12^

Authorization for the translation and adaptation of the BIT into Brazilian Portuguese was requested and approved by the research coordinator and author of the instrument, Dr. Allison Ditmars of the Indiana University School of Medicine.

Two speech therapists, native Brazilian Portuguese speakers and fluent in American English, performed the translations and the cultural adaptations of the Beginner's Intelligibility Test instrument for Brazilian Portuguese independently, considering the semantic, idiomatic, experimental, and conceptual equivalences.

After the discussion of both translations, a committee of experts met to perform the analysis and comparison of the two versions translated into Portuguese and the preliminary synthesis version in Portuguese was built. The translators involved in the process plus two evaluators with competence in healthcare and auditory rehabilitation participated in this committee. The committee compared the original version to the preliminary synthesis version and modifications were made according to the decisions taken by this committee of experts, ensuring semantic, idiomatic, conceptual, linguistic, experiential, and contextual equivalence.

The preliminary synthesis version in Portuguese obtained in the synthesis stage was sent to a fifth professional, native of the English language and fluent in Portuguese, without knowledge of the original questionnaire and not a healthcare professional, to carry out the back-translation. This phase aimed to assess whether the items reflected the content of the original version.

From the back-translation, the committee of specialists examined, compared the preliminary synthetic versions in Portuguese with the back-translated version performed necessary changes, which generated the synthetic version in Brazilian Portuguese.

For the pre-test stage, 20 children with prelingual, sensorineural, bilateral profound hearing impairment, CI users with total insertion of electrodes in the cochlea, without comorbidities, with device use time equal to or greater than 36 months were selected, who underwent surgery before the age of 7 and who participated in an auditory (re)habilitation program with an exclusively aurioral therapeutic approach.

The evaluation of the sample's speech intelligibility consisted of three stages: application of the translated version of the BIT (synthesis version), transcription of the recording of the sentences spoken by the participant, evaluation of the recording by the judges.

Each participant was asked by the research speech therapist to repeat the 10 sentences of one of the BIT lists, one by one, and the collection record was recorded in video and audio, in order to enable the analysis by the judges. The aid of pictures was also a resource used for the child to reach the target sentence. If the participant still had difficulties in understanding the sentence, the evaluator offered the support of orofacial reading (LOF). To improve and standardize the audio quality of the analyses, the functions available in the Aiseesoft Video Converter Ultimate Software were used. Thus, it was possible to eliminate the presence of noise, increase the audio volume and also standardize all analyses. All files were converted into audio, in wave format. Each participant’s file was saved in individual files and randomly selected to be presented to the judges.

Ten adult individuals participated as judges, of both sexes (50% female) with an average age of 34.3 years old and all with at least higher education and no previous experience with the speech of people with hearing impairment. The spelling transcription was requested to the judges, that is, to write what they understood from the oral emission of the recordings of two participating children, based on the audio recording of the sentences repeated by them.

In this way, each judge heard and transcribed two different children, and each of these children reproduced one of the four BIT lists. This procedure was adopted in order to ensure that there was no familiarity with the sentences analyzed by the evaluator.

At the end of each child's speech transcription, the judge was asked to classify, that is, to categorize the participant's speech intelligibility, based on the Speech Intelligibility Rating — SIR^13^ scale, as shown in the table below: (Table 1).

**Table 1:** Categories and their classification correspondences established by Allen et al. (1998)^14^ to assess speech intelligibility in children.

Category	Speech intelligibility level classification
5	Speech is intelligible to all listeners; the child is easily understood in all situations.
4	Speech is intelligible to listeners who have a little experience with hearing impaired speech; the listener does not need to concentrate too much.
3	Speech is intelligible to listeners who concentrate and do orofacial reading in a known context.
2	Speech is unintelligible; speech intelligibility corresponds to the level of single words when context and orofacial reading are available.
1	Unintelligible speech; pre-recognition of words in oral language; main way of communication in day-to-day life may be gestures.

Considering that different judges evaluated the same children, that different lists were applied and that at least two judges analyzed the recording of one of the children, two measures were randomly selected for children evaluated by more than two judges.

The scoring of the lay judges’ transcripts was performed by two other judges, speech therapists, members of the expert committee, with experience in rehabilitation of hearing disorders, both independently and blindly. The scoring is based solely on the degree of correspondence between the target sentence and the judges’ transcripts, for example, if the target sentence is: “My car is blue”, and the child says: “My car is a trunk”, it will score 3 out of 4 points. The lay judges were assured of prior and detailed knowledge of the criteria for scoring each sentence of the instrument’s lists (Table 1).

The content validation of the instrument translated and adapted to Portuguese was carried out by the committee of experts who participated in the stages of the instrument translation and analysis of the judges’ answers during the assessment of speech intelligibility with the translated BIT instrument.

### Statistical analysis

The results were statistically analyzed using the aid of the SAS software (version 9.2) and a significance level of 0.05 was determined for each hypothesis test. Cohen's Kappa study was used to determine whether there was agreement among the judges regarding the variable “Classification” of the speech intelligibility level of the analyzed children, and the Intraclass Correlation Coefficient (ICC) was used to set the variable “Score”. The relationship between “Classification” and “Score” was evaluated using Spearman’s correlation test, additionally, we sought to establish cutoff points in the variable “Score” corresponding to “Classification”, through linear regression.

## Results

The translation stage took place for the 4 lists, 40 sentences of the Beginner’s Intelligibility Test (BIT) instrument into Brazilian Portuguese, considering the semantic, idiomatic, experimental, and conceptual equivalences, and it took place without any difficulties identified by the translators. The terms used in the Portuguese language were similar and those that presented differences between the translators did not bring significant divergences to its understanding. Most were related to the use of the definite article, for example, one translator considered for the phrase “My car is blue” the translation “O meu carro é azul” in Portuguese, and the second “My car is blue” (“Meu carro é azul, in Portuguese, without the masculine definite article “O”). After the process of translation and cross-cultural adaptation, the Brazilian Portuguese version of the instrument was generated, in its synthesis version for application in the pre-test (Table 2).

**Table 2:** Application guidance and sentence lists after translation and cross-cultural adaptation of the BIT.

BIT Application Guidelines
Production and recording: The task is the repetition of the imitation in response to the model, live voice, of an examiner by the examinee. Images, objects and LOF can be used to transmit the target sentence and must be recorded in audio.
Scanning and editing: Production recordings should be edited to remove examiner models and any extraneous signals (research purpose). The sessions are digitally edited to save the examinee's production of the 10 sentences, files must be merged, and separate files created for the judgment of the hearing judges. Each sentence is heard twice by the hearing judges with a time of 4 signaled seconds between sentences.
Listening judges’ reproduction: The speech production of each child must be evaluated, and they are instructed to listen carefully to each sentence and record what they hear in cursive, guessing if necessary.
Scoring: The BIT score for each child is the score for correctly transcribed words. All words are scored, and all have equal weight. In general, because the BIT is a measure of productive speech intelligibility, the onus in scoring for conveying the linguistic message is placed on the speaker, rather than the listener. Several principles address specific points: a) All words in the target sentence must match in the transcribed response, this is, orthographic and phonological correspondence; b) If a transcript gives alternative words as answers, the word is considered unintelligible and scored as misspelled; c) No “partial credit” based on parts of words, punctuation as incorrect; d) The order of the transcribed words must be considered for the punctuation, if it is changed, it is scored as incorrect; c) Substitutions and/or inversions of words made by the judges are considered as an incorrect score; d) Some degree of dialectic and spelling variation is tolerated, e.g. for the target sentence “The grandmother falls”, both “The grandma falls” and “Granma falls” score 3 out of 3.

Lista de sentenças traduzidas – BIT	
**Lista 1**	**Lista 2**
O bebê cai.	O papai corre.
A mamãe anda.	O bebê chora.
O pato nada.	O cachorro come.
O menino senta.	A menina bebe.
A vovó dorme.	O palhaço cai.
Aquela é uma cama pequena.	Aquela é uma cama grande.
O menino andou até a mesa.	O menino andou até a cadeira.
Meu carro é azul.	A minha van é verde.
Ele está escovando os dentes.	Eles estão tocando bateria.
**Lista 3**	**Lista 4**
O papai anda.	O urso dorme.
O coelho bebe.	A mamãe senta.
O cachorro dorme.	O coelho salta.
A menina pula.	O coubói pula.
A mamãe lê.	A vovó cai.
Aquela é uma cadeira marrom.	Aquele é um chapéu preto.
O menino está em cima da mesa.	O menino está embaixo da mesa.
Meu avião é grande.	Meu avião é pequeno.
Ele está amarrando o sapato.	Ele está pintando a cadeira.
Ela está penteando o cabelo.	Ela está fazendo o jantar

The face and content validity of the translated and adapted BIT was confirmed through the understanding of each sentence of the 4 lists by the majority of CI users participating in the pre-test phase. Content validity among the members of the expert committee was unanimous for the four lists of sentences.

There was inter-evaluator reliability regarding the variable “classification” of the speech intelligibility level of the analyzed children (weighted κ = 0.95, *p* < 0.0001) and for the quantitative data of the variable “Score” (ICC = 0.979, *p* < 0.0001).

In order to establish a relationship between the “Score” given in the speech intelligibility analysis of the 10 phrases in each list and the “Classification” proposed by Allen et al.,^14^ an evaluation of the “Classification” was carried out according to the “Score”.

**Figure 1:**
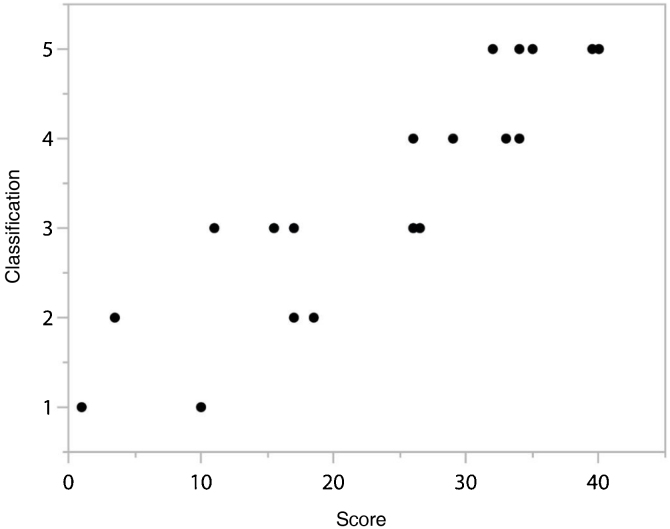
Scatter chart between “Classification” and “Score” results.

**Figure 2:**
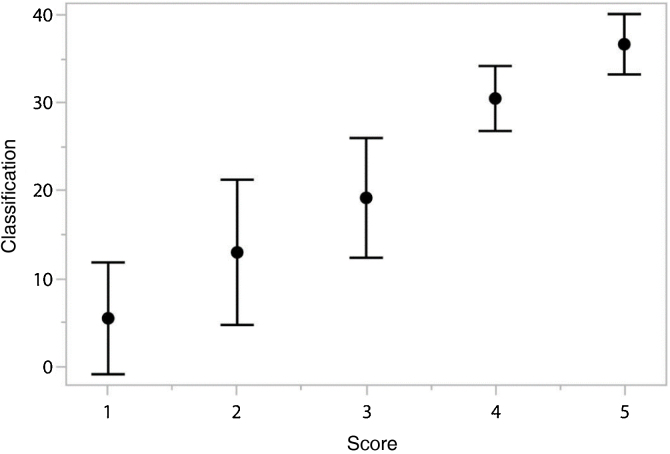
Average and standard deviation of the “Score” in the “Classification” function.

As can be seen in Figure 1, Figure 2, there was a directly proportional relationship between “Classification” and “Score” (Spearman’s Rho = 0.907, *p* < 0.0001).

**Figure 3:**
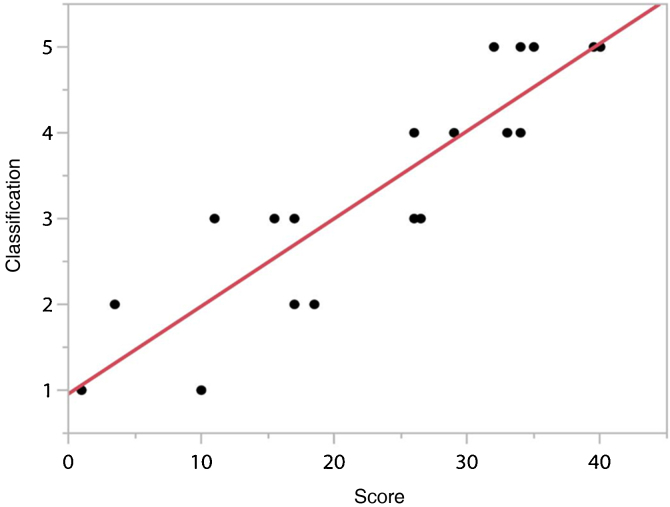
“Score” cutoff values for “Classification” of speech intelligibility level.

It was not possible to statistically determine the cutoff points of the score due to the sample “n”, however, when performing the linear adjustment between the “Classification” as a function of the “Score”, it resulted in a linear coefficient of 0.102 and intercept of 0.96. The determination coefficient (R^2^) was 0.823, showing that the linear adjustment explains 82.3% of the data variability (*p* < 0.0001), as can be seen in Fig. 3.

From the adjustment, the possible transition values (cuts) from “Score” to “Classification” were established (Table 3).

**Table 3:** Description of results, cuts for model classification, based on scores and classifications found in the studied sample (Portuguese version).

Sujeito	Pontuação	Classificação	Classificação modelo
S11	1	1	1
S18	3	2	1
S9	10	1	2
S13	11	3	2
S16	15	3	3
S12	17	2	3
S5	17	3	3
S4	18	2	3
S14	26	3	4
S20	26	4	4
S17	16	3	4
S19	29	4	4
S2	32	5	4
S10	33	4	4
S15	34	5	4
S3	34	4	4
S8	35	5	5
S6	39	5	5
S7	39	5	5
S1	40	5	5

## Discussion

In Brazil, we do not have studies adapted for the Brazilian Portuguese language that assess the speech intelligibility of children using CI. Therefore, this is the first study that performs the cross-cultural adaptation of an instrument capable of assessing speech intelligibility in children using CI.

In order to accurately define intelligibility in implanted children, the first step is to provide valid and reproducible instruments for future applications.^15^

The methodology used for translation and cultural adaptation of the Beginner’s Intelligibility Test (BIT) proved to be efficient, as seen in previous studies.^11^, ^15^, ^16^, ^17^

The Portuguese version of the Beginner’s Intelligibility Test (BIT) met the criteria of conceptual and semantic equivalence, verified both by the expert committee, which it considered adequate for the target population, as well as mentioned by the judges during the analysis of the recordings.

Thus, the face and content validity gave the instrument its semantic, idiomatic and conceptual equivalence through the expert committee and the participation of the research subjects.^10^, ^11^, ^12^

In the content analysis, the BIT proved to be relevant to its purpose, that is, to evaluate speech intelligibility in children using CI.

In this perspective, verifying whether the instrument presents inter-evaluator reliability is of fundamental importance. In this study, the reliability of the application of the test was confirmed by the inter-evaluator, both for the score of the analysis of the children's speech records and the proposed classification.

Reliability and validity are considered the main measurement properties of such instruments. Reliability is the ability to reproduce a consistent result over time and space. Validity refers to the property of an instrument to measure exactly what it proposes.^18^ Such criteria were achieved in an equivalent way in the present study, showing agreement between the judges both for the variable “classification” of the children's speech intelligibility level (Kappa weighted = 0.95, *p* < 0.0001) and for the inter-evaluator reliability analysis in relation to the speech intelligibility analysis score of the 10 sentences from a list (ICC = 0.979, *p* < 0.0001).

In the final version of the instrument (Table 2) the guidelines for its analysis were preserved, in accordance with the original version. With regard to how the instrument defines the score per subject, in the original instrument it does not have a precise definition for analysis. Thus, this score was prepared by the author, in order to facilitate the analysis in clinical practice and use it as a mediator with the Speech Intelligibility scale — SIR.

For the pre-test phase of the instrument translation process, we sought to adapt the sample “n” according to what is recommended in the literature. Although there is no evidence of a consensus between the main recommendations, an attempt was made to adapt to the most widespread recommendations so far, from COSMIN, which considers an adequate number of participants for this phase to be 30–50 subjects^19^ while others recommend the application in a “n” sample between 15 and 30 subjects.^17^

It is believed that this final translated and adapted version of the Beginner’s Intelligibility Test (BIT) will make a strong contribution to the assessment and monitoring of the development of children with hearing loss who use hearing Aid Electronic Devices (AEDs), mainly with regard to decision-making regarding the planning and conduct of the team based on the results of auditory rehabilitation and accessibility to communication.

Instruments for evaluating and classifying auditory perception and language and speech development are already well known by professionals who work with this population segment.^20^, ^21^, ^22^ A support instrument for the assessment of speech intelligibility in clinical practice becomes essential in the monitoring and adequate therapeutic planning, as well as favoring the evolution in the clinical condition of ASD users.

When analyzing the results referring to the punctuation and classification of the participating judges, we tried to verify if there was a relationship between these measures (Figure 1, Figure 2), once established, it will greatly contribute to the process of evaluating and monitoring the development of speech intelligibility of children with typical speech development or not.

The need to establish a cutoff value for the score, after analyzing the child's oral production of the BIT phrases, performed by judges to classify their speech intelligibility level (Fig. 3 and Table 4), may provide greater objectivity and effectiveness in interpreting the set of results during the evaluation or monitoring of patients in the rehabilitation process. Ensuring reliable assessment instruments and their results requires methodological accuracy and periodic reassessments, with studies on potential improvements, going through new validation stages, as it occurs a greater availability of collected data and/or changes in the profile of participants.^12^, ^23^

**Table 4:** Description of the possible cut results for model classification, based on the scores and classifications found in the studied sample.

Classification^a^	Scoring
1	0‒5
2	6‒15
3	16‒24
4	25‒34
5	35‒40

a Second category of speech intelligibility proposed by Allen et al. (1998).^14^

In the literature, some standards are already established, such as the reference by Flipsen and Colvard^24^ that indicate speech intelligibility for normal-hearing children in about 50%, around 2 years of age, and at 4 years of age, they presented 100%, that is, similar to the speech intelligibility of adults.

The performance of perception, speech production and oral language development of implanted children is considered significantly higher when compared to children using hearing aids AASI. The aurioral approach advocates the development of auditory skills in the auditory habilitation process of these children, being a determining factor in these results.^25^ Such data corroborate the present study, considering the predominance of the aurioral approach, the eligibility criteria of the participants, with the classification of the level of speech intelligibility.

Santana^26^ demonstrated in his study that children using CI can effectively acquire oral language, with more intelligible speech than those using ISAD, especially considering that the surgery was performed precociously.^27^

Regarding the results obtained through the speech intelligibility scale, the speech of ten-year-old children was classified between levels 4–5 of the scale, which represents levels corresponding to understanding by all listeners, that is, totally intelligible. Wu et al.^28^ found similar results in terms of classification, in which approximately 80% of 21 implanted children were able to reach the most advanced levels on the scale after 5 years of CI use. In the present study, the variable time of use was not considered for the analysis of the achieved classification.

Therefore, after completing all stages, it can be stated that the translation and adaptation of the original instrument in English to Portuguese was satisfactory, and that the instrument adapted to the Brazilian clinical context, is no longer a linguistic barrier. This instrument may also have the potential to become an easily accessible tool in the clinical practice of Brazilian speech therapists, as well as in English.

However, it is important to point out that studies with a larger sample are needed, which, in addition to translation and cross-cultural adaptation, will give the instrument its clinical validity, so that the effectiveness of the BIT Test in Brazil in children using CI or other electronic hearing aid device is confirmed, such as Individual Sound Amplification Devices (AASI) or Bone-Anchored Hearing Aids (PAAO), as well described by the literature.^10^, ^11^, ^12^

The results of the cross-cultural adaptation encourage us to propose a randomized study, preferably, that evaluates its applicability in children using CI, considering the variables: etiology, age at surgery, time of device use and therapeutic approach.

## Conclusion

The version adapted to the Portuguese language maintained the semantic, idiomatic, conceptual, and cultural equivalence, according to the evaluation of the expert committee and through the evaluation of the intelligibility of the children using CI, confirming the face and content validity and the reliability of the inter-evaluator analysis.

The analysis of children's oral production records of the BIT lists allows a preliminary association between the score and the classification proposed by Allen et al.^14^ and studies with a larger sample may confirm this.

## Financing

This research did not receive any specific funding from funding agencies in the public, commercial, or not-for-profit sectors.

## Conflicts of interest

The authors declare no conflicts of interest.
